# Supplementary material for: Bacterial community diversity, lignocellulose components, and histological changes in composting using agricultural straws for Agaricus bisporus production
Source: PeerJ. 2021 Feb 9;9:e10452. doi: 10.7717/peerj.10452 (PMC7879949; doi:10.7717/peerj.10452)
Supplement: Supplemental Information 1 [file peerj-09-10452-s001.docx]

**S1** The fomulation of six different AS compost

|  | **Main material** | **Rapeseed cake** | **CaSO_4_** | **CaCO_3_** | **Ca(H_2_PO4)2·H2O** | **CO(NH_2_)_2_** | **Theoretical N** | **Theoretical C/N** |
| --- | --- | --- | --- | --- | --- | --- | --- | --- |
| **WS** **compost** | 85% | 10% | 1.5% | 1.5% | 1% | 1% | 2.06 | 34.55 |
| **RS compost** | 85% | 10% | 1.5% | 1.5% | 1% | 1% | 2.25 | 30.42 |
| **CS compost** | 90% | 5% | 1.5% | 1.5% | 1% | 1% | 1.85 | 29.75 |
| **CC compost** | 83% | 12% | 1.5% | 1.5% | 1% | 1% | 2.14 | 33.32 |
| **C compost** | 95.2% | 0% | 1.5% | 1.5% | 1 % | 0.8% | 2.04 | 29.96 |
| **B compost** | 83% | 12% | 1.5% | 1.5% | 1% | 1% | 1.62 | 34.42 |
