# Supplementary figures and images for: Bacterial community diversity, lignocellulose components, and histological changes in composting using agricultural straws for Agaricus bisporus production
Source: PeerJ. 2021 Feb 9;9:e10452. doi: 10.7717/peerj.10452 (PMC7879949; doi:10.7717/peerj.10452)

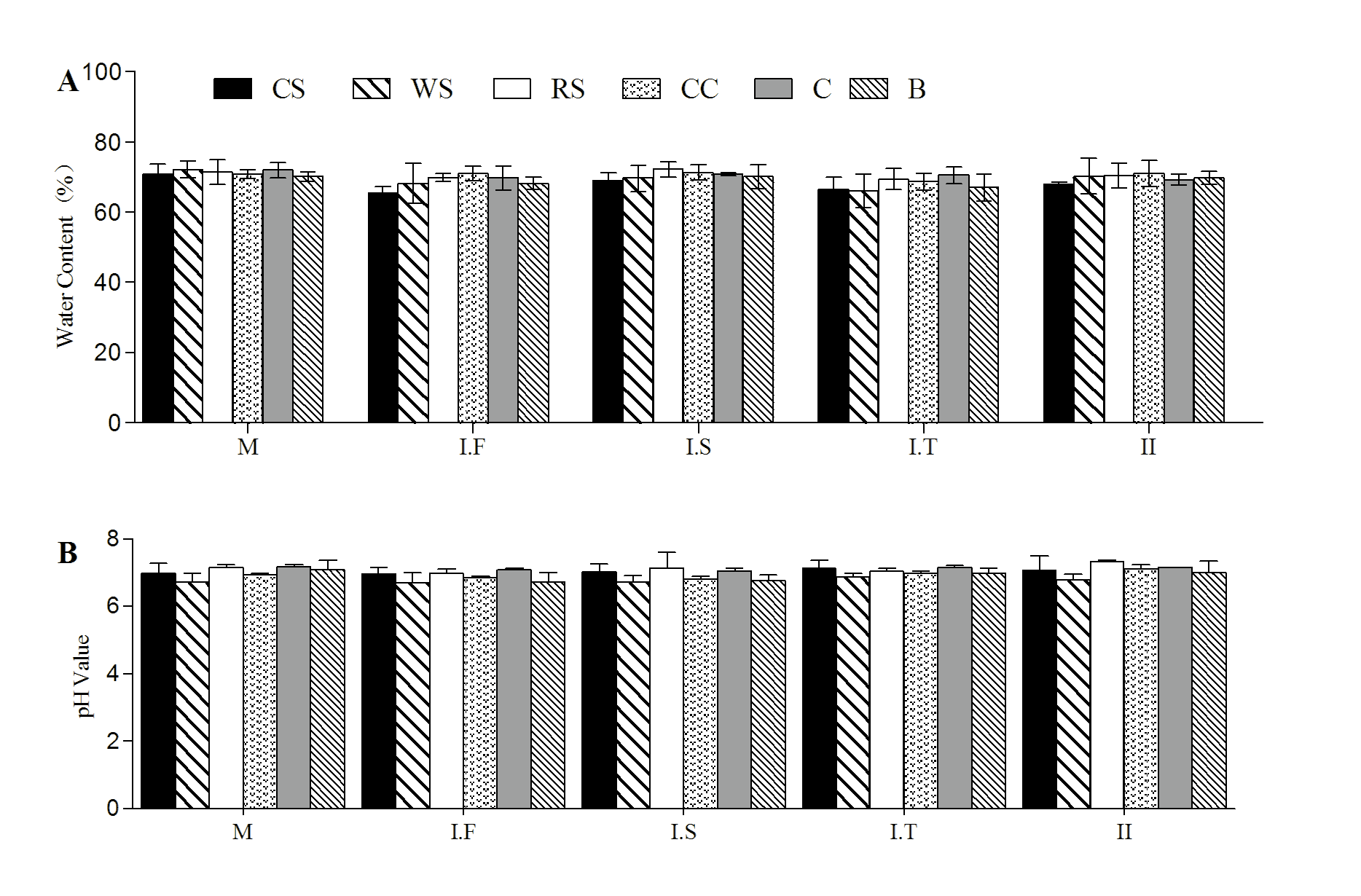

Supplement: Supplemental Information 2 [file peerj-09-10452-s002.png]

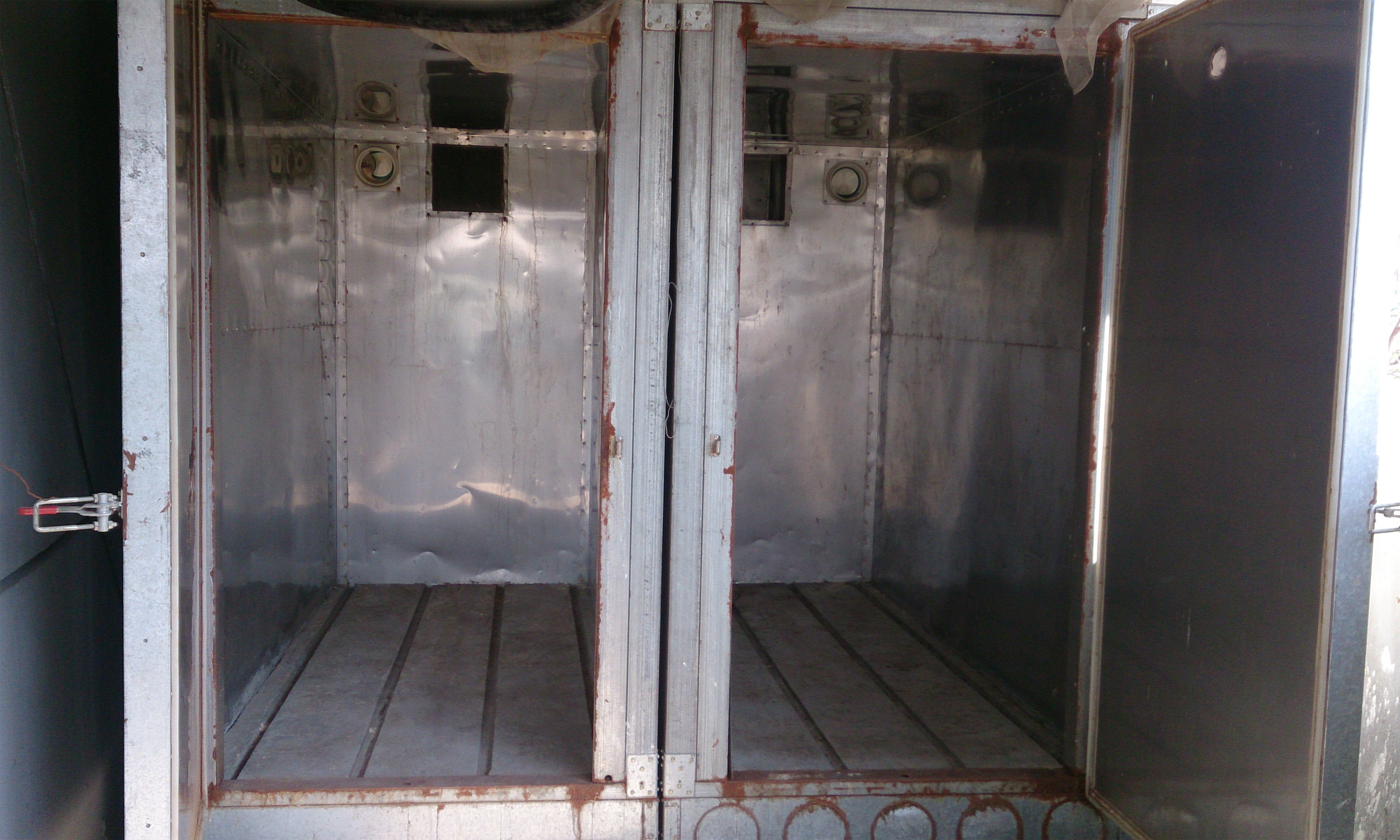

Supplement: Supplemental Information 4 [file peerj-09-10452-s004.png]
