# Supplementary material for: Bacterial community diversity, lignocellulose components, and histological changes in composting using agricultural straws for Agaricus bisporus production
Source: PeerJ. 2021 Feb 9;9:e10452. doi: 10.7717/peerj.10452 (PMC7879949; doi:10.7717/peerj.10452)
Supplement: Supplemental Information 3 [file peerj-09-10452-s003.docx]

| **Item** | **Time 2015 (Mean±SE)^a^** | | |  | **Time 2016 (Mean±SE)^a^** | | |  | **Time 2017 (Mean±SE)^a^** | | |
| --- | --- | --- | --- | --- | --- | --- | --- | --- | --- | --- | --- |
|  | TY (g/kg) | T_3_ (g/kg) | BE (%) |  | TY (g/kg) | T_3_ (g/kg) | BE (%) |  | TY (g/kg) | T_3_ (g/kg) | BE (%) |
| **CS** | 618.7±30.80^a^ | 481.5±26.62^a^ | 77.8±0.69^b^ |  | 601.4±63.34^a^ | 520.7±45.50^ab^ | 87.1±2.21^a^ |  | 570.0±23.68^a^ | 501.5±25.69^a^ | 88.4±5.49^a^ |
| **WS** | 517.7±28.51^ab^ | 381.9±25.32^b^ | 73.7±1.73^b^ |  | 609.0±25.94^a^ | 552.7±16.33^a^ | 90.9±1.63^a^ |  | 582.9±10.73^a^ | 501.2±20.17^a^ | 88.0±3.45^a^ |
| **RS** | 564.5±15.60^ab^ | 515.8±11.02^a^ | 91.4±1.33^a^ |  | 531.3±36.92^a^ | 427.9±35.13^ab^ | 80.4±1.99^ab^ |  | 565.5±37.41^a^ | 502.0±35.92^a^ | 88.7±1.97^a^ |
| **CC** | 462.4±26.60^bc^ | 354.5±4.53^b^ | 77.3±3.78^b^ |  | 531.0±41.29^a^ | 418.2±37.77^b^ | 78.8±4.27^ab^ |  | 500.8±30.21^ab^ | 365.7±35.15^b^ | 72.6±2.88^ab^ |
| **C** | 389.9±11.68^cd^ | 245.4±11.50^c^ | 63.1±1.23^c^ |  | 346.9±20.39^b^ | 231.4±7.18^c^ | 74.0±2.94^b^ |  | 374.5±36.44^bc^ | 224.1±17.05^c^ | 60.4±3.06^b^ |
| **B** | 322.4±18.57^d^ | 245.2±11.37^c^ | 76.4±2.77^b^ |  | 288.9±17.93^b^ | 223.3±12.46^c^ | 77.4±1.3^ab^ |  | 344.9±28.97^c^ | 283.2±27.96^bc^ | 82.9±7.18^a^ |
| **ANOVA^b^** | 22.95** | 44.41** | 17.25** |  | 13.12** | 22.75** | 6.04** |  | 12.96** | 19.41** | 6.55** |

**S3** Analysis of mushroom yield (*Agaricus bisporus*) cultured on six different AS-based substances in three years

^a^Data were expressed as mean ± standard error (n=10). The data in the same column marked with different small case letters are significantly

different(Tukey’s HSD, *p*<0.05).^b^ *F* value in one-way ANOVA: *p*<0.05 (*) or 0.01 (**)
