# Supplementary material for: Bacterial community diversity, lignocellulose components, and histological changes in composting using agricultural straws for Agaricus bisporus production
Source: PeerJ. 2021 Feb 9;9:e10452. doi: 10.7717/peerj.10452 (PMC7879949; doi:10.7717/peerj.10452)
Supplement: Supplemental Information 5 [file peerj-09-10452-s005.docx]

The 24 link to the16s RNA sequence data of compost using six agriculture straw as main substrate at Phase I of the 3^th^(I.F), 6^th^(I.S), 10^th^ (I.T) day, and Phase II.

1.SONG, TINGTING (2020): II.CS.fastq. figshare. Dataset. <https://doi.org/10.6084/m9.figshare.12764129.v1>

2.SONG, TINGTING (2020): II.WS.fastq. figshare. Dataset. <https://doi.org/10.6084/m9.figshare.12764126.v1>

3.SONG, TINGTING (2020): II.RS.fastq. figshare. Dataset. <https://doi.org/10.6084/m9.figshare.12764123.v1>

4.SONG, TINGTING (2020): II.CC.fastq. figshare. Dataset. <https://doi.org/10.6084/m9.figshare.12764120.v1>

5.SONG, TINGTING (2020): II.C.fastq. figshare. Dataset. <https://doi.org/10.6084/m9.figshare.12764117.v1>

6.SONG, TINGTING (2020): II.B.fastq. figshare. Dataset. <https://doi.org/10.6084/m9.figshare.12764105.v1>

7.SONG, TINGTING (2020): I.T.WS.fastq. figshare. Dataset. <https://doi.org/10.6084/m9.figshare.12764141.v1>

8.SONG, TINGTING (2020): I.T.RS.fastq. figshare. Dataset. <https://doi.org/10.6084/m9.figshare.12764081.v1>

9.SONG, TINGTING (2020): I.T.CS.fastq. figshare. Dataset. <https://doi.org/10.6084/m9.figshare.12764078.v1>

10.SONG, TINGTING (2020): I.T.CC.fastq. figshare. Dataset. <https://doi.org/10.6084/m9.figshare.12764075.v1>

11.SONG, TINGTING (2020): I.T.B.fastq. figshare. Dataset. <https://doi.org/10.6084/m9.figshare.12764069.v1>

12.SONG, TINGTING (2020): I.T.C.fastq. figshare. Dataset. <https://doi.org/10.6084/m9.figshare.12764072.v1>

13.SONG, TINGTING (2020): I.S.WS.fastq. figshare. Dataset. <https://doi.org/10.6084/m9.figshare.12764057.v1>

14.SONG, TINGTING (2020): I.S.RS.fastq. figshare. Dataset. <https://doi.org/10.6084/m9.figshare.12764054.v1>

15.SONG, TINGTING (2020): I.S.CS.fastq. figshare. Dataset. <https://doi.org/10.6084/m9.figshare.12764048.v1>

16.SONG, TINGTING (2020): I.S.CC. figshare. Dataset. <https://doi.org/10.6084/m9.figshare.12764042.v1>

17.SONG, TINGTING (2020): I.S.C.fastq. figshare. Dataset. <https://doi.org/10.6084/m9.figshare.12764036.v1>

18.SONG, TINGTING (2020): I.S.B.fastq. figshare. Dataset. <https://doi.org/10.6084/m9.figshare.12764030.v1>

19.SONG, TINGTING (2020): I.S.B.fastq. figshare. Dataset. <https://doi.org/10.6084/m9.figshare.12764030.v1>

20.SONG, TINGTING (2020): I.F.WS.fastq. figshare. Dataset. <https://doi.org/10.6084/m9.figshare.12764018.v1>

21.SONG, TINGTING (2020): I.F.RS.fastq. figshare. Dataset. <https://doi.org/10.6084/m9.figshare.12764012.v1>

22.SONG, TINGTING (2020): I.F.C.fastq. figshare. Dataset. <https://doi.org/10.6084/m9.figshare.12763997.v1>

23.SONG, TINGTING (2020): I.F.B.fastq. figshare. Dataset. <https://doi.org/10.6084/m9.figshare.12763949.v1>

24.SONG, TINGTING (2020): I.F.CC.fastq. figshare. Dataset. <https://doi.org/10.6084/m9.figshare.12764024.v1>
